# The Microbiome in Systemic Sclerosis: Pathophysiology and Therapeutic Potential

**DOI:** 10.3390/ijms232416154

**Published:** 2022-12-18

**Authors:** Suhee Kim, Hee Jin Park, Sang-Il Lee

**Affiliations:** Department of Internal Medicine and Institute of Health Science, College of Medicine, Gyeongsang National University and Hospital, Jinju 52727, Republic of Korea

**Keywords:** systemic sclerosis, microbiota, animal model, bioavailability, oral drugs

## Abstract

Systemic sclerosis (SSc), also known as scleroderma, is an autoimmune disease with unknown etiology characterized by multi-organ fibrosis. Despite substantial investigation on SSc-related cellular and molecular mechanisms, effective therapies are still lacking. The skin, lungs, and gut are the most affected organs in SSc, which act as physical barriers and constantly communicate with colonized microbiota. Recent reports have documented a unique microbiome signature, which may be the pathogenic trigger or driver of SSc. Since gut microbiota influences the efficacy and toxicity of oral drugs, evaluating drug–microbiota interactions has become an area of interest in disease treatment. The existing evidence highlights the potential of the microbial challenge as a novel therapeutic option in SSc. In this review, we have summarized the current knowledge about molecular mechanisms of SSc and highlighted the underlying role of the microbiome in SSc pathogenesis. We have also discussed the latest therapeutic interventions using microbiomes in SSc, including drug–microbiota interactions and animal disease models. This review aims to elucidate the pathophysiological connection and therapeutic potential of the microbiome in SSc. Insights into the microbiome will significantly improve our understanding of etiopathogenesis and developing therapeutics for SSc.

## 1. Introduction

Systemic sclerosis (SSc) is an autoimmune disease characterized by substantial inflammation, vasculopathy, and fibrosis, affecting the skin and internal organs [1]. Although diverse cellular and molecular mechanisms involved in SSc have been demonstrated, its etiology and pathogenesis are not clearly understood. SSc is a rare but deadly rheumatologic disease [2]. Effective disease-modifying therapies for SSc are still unavailable owing to the complexity and heterogenicity of the pathological and clinical features.

The gastrointestinal tract (GIT) is the second most affected organ in SSc, following the skin [3], and interstitial lung disease (ILD) is a substantial cause of mortality [4]. Several recent studies have highlighted the association between SSc and the microbiome of organs, including dysregulated microbiota and related metabolites in patients with SSc [5,6]. This impaired microbiome homeostasis supports the concept of microbiome targeting as a treatment option for SSc. Although the exact mechanisms involved in the initiation of SSc are unclear, immune activation has been postulated as a critical event leading to fibrosis [1]. Considering the immunological importance of the fibrotic process of SSc, understanding the regulatory effect of the microbiome on the immune system would be essential in unraveling novel therapeutic targets and disease management.

The microbiome plays a vital role in maintaining the immune system [7] by consistently interacting with host immune cells [8]. For example, dendritic cells (DCs) can directly collect antigens from a resident or invading microorganism [7] and acquire appropriate signals for anti- or pro-inflammatory responses depending on the antigen source [9,10]. Activated DCs regulate the differentiation of T cells into effector helper T (Th) or regulatory T (Treg) cells [7]. DCs also promote the release of immunoglobulin A (IgA) from B cells, which prevents pathogenic bacteria from adhering to the epithelium and neutralizes the bacteria [8]. On the other hand, the regulatory role of commensal bacteria on host immune cells has also been demonstrated [9]. Polysaccharide A, a molecule of the commensal *Bacteroides fragilis*, enhances interleukin-10 (IL-10) production by Treg cells by binding to toll-like receptor 2 (TLR2) on the surface of plasmacytoid DCs (pDCs) during gut inflammation [11]. *Clostridium* strains preferentially facilitate the expansion of colonic Treg cells [12] and *Clostridium* (*C.*) *butyricum* is involved in their polarization into IL-10-producing macrophages [13]. Short-chain fatty acids (SCFAs), which are fermented metabolites produced by colonic microbiota, promote differentiation into Treg cells, induce the production of anti-inflammatory mediators, inhibit inflammatory responses, and maintain the integrity of the epithelial barrier [7,14,15]. Therefore, similar to other autoimmune diseases, microbiome disruption might be closely correlated with immune-related pathological events which may trigger or contribute to SSc development [16].

This review summarizes current reports on molecular mechanisms underlying SSc pathogenesis and the novel findings regarding the effects of the microbiome on SSc. We have also discussed the rationale and clinical evidence related to the microbiome challenge as a potential therapeutic option for SSc. This review also discusses a possible experimental mouse model of SSc for determining microbial-related mechanistic clues and preclinical evidence in SSc. Microbiome studies will improve the in-depth understanding of the pathophysiological mechanisms of SSc and help in the development of novel therapeutic options.

## 2. Pathogenesis of SSc

**Figure 1:**
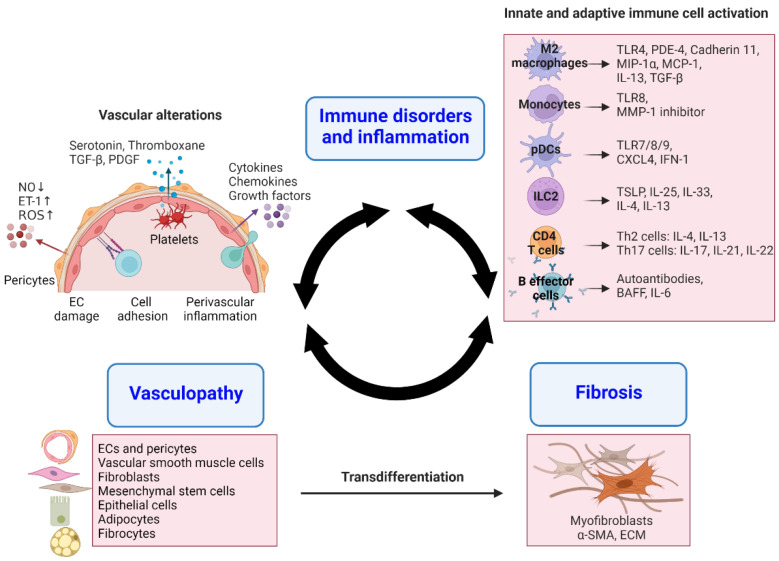
Pathological processes involved in SSc. The main pathophysiological hallmarks of SSc are immune disorders, vasculopathy, and fibrosis in tissues and organs. Diverse innate and adaptive immune cells are involved in EC activation and fibrosis. Vascular alterations and trans-differentiation toward myofibroblasts contribute to inflammation and fibrosis in the perivascular space and surrounding tissues.

The classical progression of SSc includes aberrant immunological responses, which cause inflammation and vasculopathy, eventually leading to fibrosis [1]. However, the trigger for the initiation and acceleration of the series of pathogenic processes remains unknown. Immune cells, endothelial cells (ECs), and fibroblasts are the primary cell types involved in SSc pathogenesis [1]. Therefore, it is necessary to elucidate the interplay between these cell types to inhibit the cascade of events leading to the development of SSc. The pathogenesis of SSc is summarized in Figure 1.

### 2.1. Immune Aberrations and Inflammation

Although fibrosis is the endpoint in SSc, inflammation is commonly observed as the condition progresses [1]. An inflammatory process characterized by the infiltration of immune cells may be crucial in the initiation and progression of both vasculopathy and fibrosis in response to homeostatic disturbances [17]. In the early phase of SSc, immunological components play a pivotal role in EC and vascular function. Anti-endothelial cell autoantibodies (AECA), anti-endothelin-1 type A receptor (ETAR) autoantibody, anti-angiotensin II type I receptor (AT1R) autoantibody, γ/δ T cells, cytotoxic CD4^+^ T cells (CD4^+^ CTLs), and natural killer (NK) cells are involved in EC activation and damage in SSc [17]. The infiltrates of monocytes/macrophages and T cells are detectable in inflammatory perivascular lesions at the early stages of SSc [18,19]. Furthermore, chemokines including macrophage inflammatory protein-1α (MIP-1α), monocyte chemotactic protein 1 (MCP-1 or CCL2), and IL-8 can attract more immune cells toward the inflamed lesions and contribute to the local fibrosis through overproduction of fibrogenic mediators, such as transforming growth factor-β (TGF-β) and IL-6 [20]. In this chapter, we will describe immune cells involved in the inflammatory and fibrotic processes of SSc.

Innate immune cells can initiate and amplify inflammatory events by sensing homeostatic perturbations and tissue injury [21]. Activation of innate immune cells involves the recognition of pathogen-associated molecular patterns (PAMPs) or damage-associated molecular patterns (DAMPs) via pattern-recognition receptors (PRRs). Interestingly, PRR is also expressed by epithelial, endothelial, and stromal cells, including fibroblasts, that generate and respond to inflammatory components. Among PRRs, toll-like receptors (TLRs), which are the first line of defense against pathogenic bacteria and host endogenous danger signals, are the key molecules in the pathogenesis of SSC [19]. A rare polymorphism in TLR2 was associated with anti-topoisomerase antibody positivity and enhanced IL-6 and TNF-α production by DCs [22]. TLR4, expressed in macrophages and fibroblasts, mediates fibroblast activation by recognizing fibronectin extracellular domain A (FN^EDA^) on fibroblasts in SSc [23,24]. Furthermore, TLR8 stimulation can contribute to matrix deposition by enhancing the activity of tissue inhibitor of matrix metalloproteinase (MMP)-1 in monocytes in patients with SSc [25]. Additionally, TLR7, TLR8, and TLR9 on pDCs enhance type I interferon (IFN-1) by sensing DNA/RNA shuttled by autoantibodies or CXC chemokine ligand 4 (CXCL4) into endosomes [26,27]. TLR-mediated innate immune signaling is involved in persistent fibrotic activation in SSc [28]. The downstream pro-inflammatory cytokines of TLR signaling, IL-1 and IL-6, are critical molecules involved in innate immune signaling pathways in SSc [29].

Gene expression analysis from SSc-affected tissues revealed an extensive network of immune-fibrotic genes representing an innate immune signature indicative of IFN and M2 macrophage activation [30,31]. In SSc, circulating monocytes and tissue macrophages differentiate into M2 macrophages (expressing CD163 and CD204) under the influence of type 2 cytokines, such as IL-4 and IL-13 [17]. Elevation of soluble CD163 was observed in the blood of patients with SSc [32]. The release of profibrotic mediators from M2 macrophages is mediated by phosphodiesterase (PDE)-4. The inhibition of PDE-4 reduced the release of profibrotic cytokines from M2 macrophages and fibroblast activation in a preclinical SSc model [33]. Another key molecule in macrophages, cadherin 11, regulates TGF-β production [34]. Myeloid DCs (mDCs) and pDCs may play a vital role in inflammatory and fibrogenic processes in SSc by releasing IFN-1 [27,35]. Particularly, pDC induced the secretion of IFN-α and CXCL4 in the skin or blood of patients with SSc [27]. Innate lymphoid cells (ILCs) have been recently described but their profile in SSc is not well defined. ILC2 contributes to enhanced production of Th2 cytokines (IL-4 and IL-13) and extracellular matrix (ECM) deposition in response to alarmins such as IL-25, IL-33, and thymic stroma lymphopoietin (TSLP) [17]. ILC2 is elevated in the skin and correlates with the extent of skin fibrosis in SSc [36]. 

The adaptive immune system is crucial in the initiation and development of SSc [37]. T cells contribute to the activation of macrophages, ECs, and fibroblasts through cytokine production [38]. T cells have highly heterogenic types [17] and are enriched in the skin and lungs in the early stage of SSc [39]. Paleja et al. proposed an overall dysregulated immune architecture dominated by highly differentiated and chronically stimulated T cells in patients with SSc by analyzing the systemic immunome of their peripheral blood [40]. IL-4- or IL-13-producing CD4^+^ Th2 and type 2 CD8^+^ T cells (Tc2) affect fibrosis by enhancing ECM production and polarization toward M2 macrophage [38,41]. The Th17 cells producing IL-17, IL-21, and IL-22 are also abundant in the skin and peripheral blood of patients with SSc and may participate in the inflammatory process [42,43,44]. Despite contradictory results on Tregs, most of the studies reported decreased frequencies and impaired function of circulating Treg cells, and a Treg/Th17 imbalance with reduced serum TGF-β1 and IL-10 levels in patients with SSc [45,46,47]. Loss in the number and function of Treg cells could be the result of their trans-differentiation towards a Th2- and Th17-like phenotype, suggesting the contribution of Tregs in SSc pathogenesis [47]. Pro-inflammatory cytokines IL-6 and IL-1β can be involved in the conversion of Tregs to Th17 cells [48]. Tregs from the skin of patients with SSc significantly produced Th2 cytokines, such as IL-4 and IL-13. IL-33, upstream of IL-13, might stimulate the trans-differentiation of skin Tregs into Th2-like phenotypes [49]. Future studies are needed to clarify the role of Tregs in SSc development since much controversy remains about their role in SSc pathogenesis. Meanwhile, CD4^+^ CTLs and CD8^+^ CTLs present in the skin may induce EC apoptosis resulting in vasculopathy [50,51]. Patients with SSc had higher levels of follicular T cells (TFH) in the peripheral blood and skin, which induced the differentiation of B cells, resulting in the secretion of immunoglobulins through IL-21 production [52]. 

Dysregulated homeostasis of B cells plays a potential role in the pathogenesis of SSc by infiltrating the skin and lungs and producing cytokines and autoantibodies, such as IL-6, TGF-β, AECA, or anti-fibroblast autoantibody (AFA) [37]. Patients with SSc had decreased B regulatory cells (Breg) and functionally impaired IL-10 production [53], with an increased level of the B cell-activating factor (BAFF) and IL-6 production [54]. The elevated serum BAFF level was positively correlated with the severity of skin fibrosis in patients with SSc [54]. BAFF suppressed Breg cells while increasing B effector cells [55]. Because BAFF mRNA is upregulated in the patient skin samples during the early phase of SSc [54], B cells may be involved in SSc initiation before the development of fibrosis [37]. Moreover, a network between B cells and fibroblasts in SSc has been demonstrated [56]. IL-6 and TGF-β, profibrotic cytokines produced by activated B cells in SSc, can promote collagen production by directly activating fibroblasts in a contact-dependent manner in vitro [57,58]. B cells can stimulate fibroblasts by producing autoantibodies against the platelet-derived growth factor (PDGF) receptor and MMP-1, indicating the profibrotic function of B cells in SSc pathophysiology [59,60].

### 2.2. Endothelial Cells Damage and Vasculopathy

Vascular alterations are considered a key feature of SSc as they are present in almost all patients with SSc and occur in the early phases of the disease before the appearance of symptoms [61,62]. Vasculopathy includes EC damage, increased expression of adhesion molecules, increased release of growth factors, ischemia/reperfusion injury, intimal hyperplasia/proliferation, and proteoglycan accumulation [62]. Platelets, pericytes, and keratinocytes in conjunction with vascular wall cells and fibroblasts also contribute to vasculopathy [17].

EC damage and dysfunction are significant events in SSc vasculopathy. The imbalance in vasodilators and vasoconstrictors, such as decreased nitric oxide (NO) and increased ET-1 production, may lead to ischemia/reperfusion injury and subsequent oxidative stress, which influences EC [17]. Sustained EC injuries and apoptosis trigger the opening of endothelial junctions, expression of adhesion molecules, recruitment of inflammatory cells, and microvascular leakage [62]. Additionally, platelet activation, increased aggregation, and concomitant endothelial dysfunction have been observed in patients with SSc [63]. Activated platelets participate in vascular injury and fibrosis by releasing vasoconstrictors (serotonin and thromboxane) and profibrotic mediators (TGF- β, PDGF, and CXCL4) [64]. Serotonin released by platelets induced ECM synthesis in fibroblasts via activation of the TGF-β signaling pathway [65]. In vitro, platelet activation promoted the production of TSLP by dermal Ecs in an IL-1β-dependent mechanism. TSLP expression in dermal cells was correlated with the severity of skin fibrosis in SSc [66]. Ecs activated by inflammatory mediators may also affect resident or recruited cells in tissues and organs by promoting further production of cytokines and growth factors, such as IL-1, IL-33, and TSLP, in the interaction with innate immune cells, fibroblasts, and adipocytes [17]. 

Trans-differentiation of injured ECs towards myofibroblasts, referred to as the endothelial–mesenchymal transition (EndMT), may contribute to vessel wall fibrosis. During the EndMT, ECs lose the expression of EC markers including CD31, von Willebrand factor, Tie-1, and Tie-2 receptors, as well as vascular endothelial cadherin (VE-cadherin). At the same time, ECs acquire the expression of mesenchymal markers including α-smooth muscle actin (α-SMA), smooth muscle 22 (Sm22), vimentin, CD44, fibroblast-specific protein-1/S100A4, neuronal-(N-) cadherin, and collagen [67]. ECs undergo typical fibroblast-like morphological changes including loss of polarity, acquisition of elongated phenotypes, as well as proliferative, migratory, and invasive properties [68]. Eventually, the EndMT process may lead to vascular destruction, rarefaction, and fibrosis by facilitating the disaggregation and invasion of ECs, and the concomitant formation and accumulation of myofibroblasts into the perivascular space and surrounding tissues [67,69]. In addition to ECs, vascular wall-resident cells (pericytes and smooth muscle cells) appear to be trans-differentiated into myofibroblasts, contributing to SSc-related vasculopathy and fibrosis [69,70]. TGF-β and PDGF-BB released from injured ECs promote pericyte proliferation and phenotypic switching of pericytes towards myofibroblasts [67]. 

TGF-β is the most crucial cytokine associated with fibrosis and is a critical inducer of the EndMT, promoting collagen deposition and suppressing the expression of metalloproteinases [71]. TGF-β/small mothers against decapentaplegic homolog (Smad) signaling has been extensively described in SSc. TGF-β isoforms bind to the TGF-β receptor type II (TβRII) that promotes the activation of TβRI, thereby inducing cell signals via the phosphorylation of Smad proteins [72]. TGF-β1 is the major driver of pathologic fibrosis concerning the EndMT. In addition to TGF-β, possible mediators of the EndMT include Wingless/integrated (Wnt) proteins, ET-1, IL-1, TNF-α, IFN, MicroRNA (miR), reactive oxygen species (ROS), and the hypoxia-inducible factor (HIF) [67].

### 2.3. Fibroblast Activation and Extracellular Matrix Deposition

Immunological and vascular alterations drive the fibrotic processes which characterize the final stage of SSc [37]. Myofibroblasts are the critical effector cells responsible for fibrosis and collagen deposition in tissues. Fibroblasts are mesenchymal-derived cells that are often metabolically inactive [29]. However, tissue injury or inflammation induces the activation and transformation of quiescent fibroblasts into myofibroblasts that can accumulate stress fibers, α-SMA, and ECM proteins in response to tissue injury [1]. ECM-producing activated fibroblasts or myofibroblasts can be generated by the activation and trans-differentiation of a variety of cells such as resident fibroblasts, mesenchymal stem cells, endothelial and epithelial (keratinocytes) cells, pericytes, adipocytes, and circulating fibrocytes (monocytes) [73,74]. Myofibroblasts are involved in sustained organ damage and remain persistent under fibrotic conditions such as SSc [29]. Furthermore, SSc dermal fibroblasts can maintain a Th2-skewed state by suppressing the expression of the Th1 cytokine IFN-γ in skin-infiltrating CD4^+^ T cells through the overproduction of galectin-9, which negatively regulates Th1/Th17 cells [75].

The fibrotic response is characterized by a type 2-like milieu governed by IL-4- or IL-13-producing Th2-like T cells, ILC2, and M2 macrophages [17]. TGF-β is a component responsible for fibrosis and a potent inducer of fibroblast proliferation, migration, and differentiation, thereby enhancing ECM production [76]. Connective tissue growth factor (CTGF) interacts with TGF-β to sustain persistent ECM synthesis [77]. Additionally, PDGF, IL-6, Wnt/β-catenin, and Hedgehog signaling are essential drivers of fibrotic signaling responses [78]. 

Recent studies investigating gene signatures from skin biopsies of patients with SSc revealed inflammatory, fibroproliferative, limited, and normal-like subsets, indicating heterogeneous mechanisms leading to fibrosis [79,80,81,82]. The transcription factor PU.1, a fundamental regulator of the profibrotic program, was recently shown to induce the transcription of profibrotic genes and a phenotypic transition to fibrotic fibroblasts [83].

### 2.4. Pro-Inflammatory and Profibrotic Mediators in SSc

IFN-1 is an important regulator of the innate immune system [84]. Recent studies have provided evidence of a prominent IFN signature in SSc [85]. Increased expression of IFN-regulated genes in peripheral blood cells was observed in approximately half of the patients with SSc [86]. Additionally, activation of IFN-1 was observed in the serum of patients with SSc, and IFN-α production was primarily induced by pDCs [87]. IFN regulatory factors (IRF) 5, IRF7, and IRF8 regulate the expression of IFN and IFN-inducible genes in SSc [88]. A critical role of TLR-mediated IFN-1 activation has been suggested in increasing the inflammatory potential of fibroblasts [89]. 

Endogenous danger signals are called alarmins or DAMPs [90]. Alarmins are passively or actively released from cells in the microenvironment in response to cell injury, death, or immune induction [91]. They can activate both innate immune (macrophages, DCs, and neutrophils) and non-immune cells (epithelial cells, ECs, and fibroblasts), which in turn recruit inflammatory cells and activate adaptive immune responses following the production of cytokines and chemokines. Alarmins can be classified as follows: (1) nuclear, including high-mobility group box-1 (HGMB-1) and IL-1 family (IL-1α and IL-33); (2) granule derived, including α- and β-defensins, cathelicidin, and granulisin; and (3) cytoplasmic, including heat-shock and S100 proteins [90]. The serum HMGB-1 level was increased in patients with SSc, which correlated with skin thickness score and pulmonary function [92]. Activated platelets release alarmins, such as HMGB-1 and microparticles, in the serum, which are involved in endothelial damage in SSc [93]. In SSc, skin Treg cells produce IL-4 and IL-13, and peripheral blood Treg cells express Th2-like skin-homing receptors. Skin-resident Tregs expressed the ST2 chain of the IL-33 receptor and IL-33 differentiated skin Tregs into a Th2-like phenotype [49]. IL-33 might be responsible for tissue-localized dysfunction of Treg cells in SSc.

The IL-1 family is composed of 11 cytokines, including IL-1α, IL-1β, IL-18, and IL-33. A recent study reported the abnormal expression of most of the IL-1 family cytokines in autoimmune diseases including SSc. Several studies have demonstrated the potential role of IL-1 in inducing differentiation and longevity of myofibroblast in SSc [94]. IL-1α, IL-1β, and IL-18 were significantly upregulated in skin lesions of patients with SSc, and the elevation of IL-1β, IL-18, and IL-33 was observed in the serum or bronchoalveolar lavage fluid (BAL) [95,96,97]. IL-1α and IL-1β induced fibroblast proliferation, myofibroblast activation, and collagen production by promoting the production of IL-6, PDGF, and TGF-β1 in SSc or fibrosis [94,98]. Moreover, IL-1α and IL-1β enhanced the viability of SSc and normal fibroblasts in culture with the induction of N-cadherin and α-SMA expression [99]. IL-1β has also been shown to participate in the EndMT [100] and the differentiation of Th17 cells [101]. 

IL-33 is mainly produced by ECs, fibroblasts, epithelial cells, monocytes, and macrophages [102]. At the later stage of SSc, IL-33 was found constitutively in most ECs [103] and an increased level of serum IL-33 positively correlated with tissue fibrosis and vascular involvement [104,105]. IL- 33 induces fibrosis by participating in type 2 immunity, such as the polarization of M2 macrophages and proliferation of ILC2s [106,107]. In addition to its role as a traditional cytokine, IL-33 can also function as an alarmin which is released from damaged/dead cells or immune cells [90,102]. IL-33 mediates biological effects by interacting with innate immune cells (DCs, macrophages, NK cells, eosinophils, basophils, and neutrophils) expressing the ST2, a member of the TLR/IL-1 superfamily. IL-33/membrane-bound receptor (ST2L) signaling contributes to the survival and expansion of ST2^+^ cells and the production of Th2 cytokines (IL- 4, IL-5, and IL-13) [90].

IL-13, primarily produced by activated CD4^+^ Th2 cells, is a critical Th2 cytokine in fibrotic disease including SSc [108]. Although IL-13 and IL-4 are functionally similar, IL-13 appears to be a dominant cytokine in fibrosis [109]. IL-13 promotes fibrosis by driving ECM accumulation and macrophage recruitment [109]. Specifically, IL-13-producing-CD8^+^ T cells expressed skin-homing receptors in the peripheral blood of patients with SSc with infiltration in skin lesions [110]. IL-13 was suggested to be a downstream mediator of IL-33 [106].

IL-6 is a powerful mediator leading to fibrosis in SSc via the induction of B and T cell proliferation, fibroblast activation, and collagen production [111]. Significant amounts of IL-6 were produced in the peripheral blood mononuclear cells and cultured dermal fibroblasts from patients with SSc [111,112]. Patients with SSc showed elevated serum IL-6 levels, which correlated with the extent of cutaneous affection [113].

An increase in Th17 cell-derived IL-17 has been observed in the serum of patients with SSc, suggesting the relation to Th17 pathway-induced immune activation in SSc pathogenesis [38]. TGF-β, IL-21, IL-6, and IL-1β are critical mediators in the differentiation of Th17 cells producing IL-17 in response to TGF-β and IL-23 [38]. The pro-inflammatory and profibrotic properties of IL-17A have been indicated in SSc [114,115,116]. IL-17A can participate in inflammatory responses by promoting the production and expression of CCL2, IL-8, IL-6, MMPs, and adhesion molecules in dermal fibroblasts and ECs [117]. IL-17A derived from sera of patients with SSc induced EC inflammation by up-regulating chemokines (CCL-20 and CXCR4) and adhesion molecules (ICAM-1 and VCAM-1) [118], and caused the proliferation, migration, and collagen synthesis in dermal vascular smooth muscle cells (DVSMCs) [119]. In mouse models of SSc, the knockdown of IL-17A attenuated skin and lung fibrosis [116]. Additionally, IL-17A exerted profibrotic function with the expression of TGF-β, CTGF, and collagen in dermal fibroblasts [115] and the promotion of collagen deposition and epithelial–mesenchymal transition in alveolar epithelial cells [114].

## 3. Link between SSc Pathophysiology and the Microbiome

Macrophages and DCs, which are vital innate immune players of SSc, recognize pathogenic microbes and can initiate a signal transduction cascade that triggers or sustains the development of SSc. Particularly, the epithelial or mucosal barriers, where the microbiota resides, including the skin, lungs, and GIT, are prominently affected in SSc. Therefore, microbial dysbiosis potentially contributes to multiple organ dysfunction by severely influencing immune responses in SSc. Although whether the microbiome alterations observed in SSc are a primary cause or a consequence of organ pathology is not yet defined, this review provides an overview of the SSc microbiome to elucidate the potential role of the microbiome on SSc pathophysiology.

### 3.1. Gut Microbiome in SSc

#### 3.1.1. Pathogenesis of GIT Involvement in SSc

The GIT is the most affected internal organ in patients with SSc. GIT involvement occurs in more than 90% of patients during the disease course, with manifestations including constipation, diarrhea, malnutrition, gastrointestinal bleeding, and fecal incontinence [120]. As GIT involvement is observed in the early phase of SSc [121], the GIT microbiome and associated manifestation may be key factors in the etiology, pathogenesis, or progression of SSc [122]. Studies focused on GIT in patients with SSc to elaborate on the role of the microbiome on GIT complications are currently ongoing [123]. The clinically observed associations between gut microbiota and GIT symptoms may support the rationale for gut bacterial targeting as a therapeutic intervention to mitigate GIT symptoms in patients with SSc [124].

**Figure 2:**
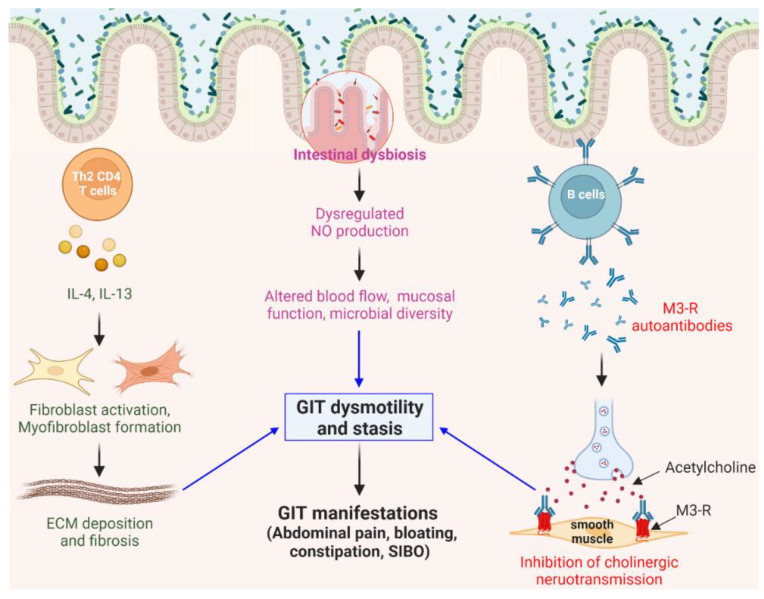
Proposed mechanism of SSc-associated gastrointestinal involvement. GIT involvement of SSc is likely associated with neuropathy and myopathy caused by the activation of the immune system. Th2 CD4 T cells induce myofibroblast formation and subsequent perivascular and interstitial fibrosis, affecting GIT dysmotility. Anti-muscarinic receptor (M_3_-R) autoantibodies can reduce GIT contractility by interfering with the acetylcholine binding on intestinal smooth muscle. Additionally, dysbiosis of commensal gut microbiota, which may regulate NO production, may influence blood flow, mucosal integrity, and GIT motility. Diminished peristalsis and luminal content stasis may be involved in the development of GI manifestations in SSc.

The pathogenic background underlying GIT involvement is likely associated with neuropathy and myopathy attributed to the activated immune system, EC damage, and vasculopathy [125] (Figure 2). Th2 CD4^+^ T cells participating in fibroblast activation can lead to perivascular and interstitial fibrosis in the GIT [125]. Anti-muscarinic receptor autoantibodies produced from B cells block cholinergic nerve stimulation (neuropathic damage) and reduce the contractility of intestinal smooth muscle (myopathy) [126]. In patients with SSc, ischemia-induced neuronal damage and collagen accumulation may result in neural dysfunction and compression in the GIT [127]. The fibrosis and atrophy of smooth muscle can cause diminished peristalsis and subsequent abdominal pain, bloating, and constipation, which often signifies an increase in gut-related morbidity and mortality. The GIT dysmotility and luminal content stasis can promote small intestinal bacterial overgrowth (SIBO), leading to malnutrition and diarrhea in patients with SSc [120,128,129]. 

Dysregulated intestinal homeostasis is closely associated with pathological events in SSc. Commensal gut bacteria may potentially influence mucosal integrity and GIT motility by regulating NO production via the denitrification pathway [130]. Wound tissue of germ-free (GF) mice highly expressed M2 macrophage-related genes [131]. Antibiotic-induced gut dysbiosis led to airway inflammation and polarization toward M2 macrophages in the lungs [132]. Considering that gut microbiota affects the pathogenesis of various autoimmune diseases, studying the influence of the gut microbiome on SSc pathophysiology is extremely important.

#### 3.1.2. Characterization of the SSc Gut Microbiome

**Figure 3:**
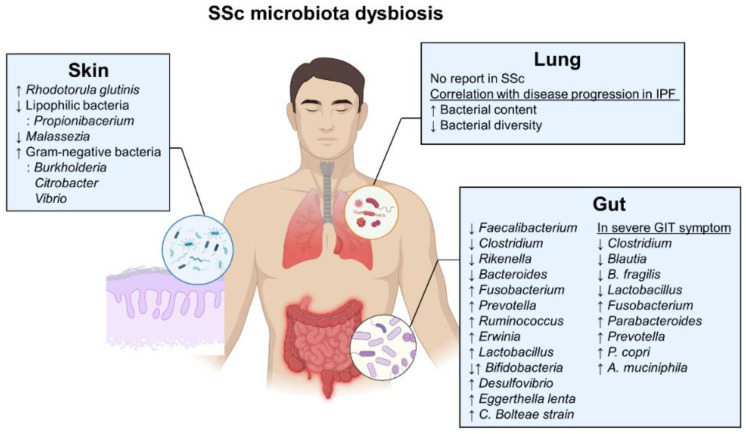
Microbiota alterations in the gut, skin, and lungs of patients with SSc.

Recent reports have demonstrated a unique gut microbial signature in patients with SSc, which is characterized by distinct alterations in the phylum (Bacteroidetes) and genus (such as *Bacteroides*, *Faecalibacterium*, *Clostridium*, *Fusobacterium*, *Prevotella*, and *Lactobacillus*) levels [5,133,134] (Figure 3). Colonic lavage from 17 patients with SSc showed a reduction in commensal genera (such as *Faecalibacterium, Clostridium*, and *Rikenella*) and an increase in pathobiont bacteria (including *Fusobacterium, Prevotella*, and *γ-Proteobacteria*) compared to 17 healthy controls (*q* < 0.1) [134]. Similarly, the fecal microbiome of patients with SSc from two independent cohorts (17 patients/cohorts) showed a significant decrease in beneficial genera (*Bacteroides*, *Faecalibacterium,* and *Clostridium*) with a decrease in phylum Bacteroidetes and an increase in harmful genera (*Fusobacterium*, *Ruminococcus*, and *Erwinia)* [6]. Butyrate-producing bacteria that play a protective role were depleted in the gut microbiome of patients with SSc (*n* = 90) and IgG4-related fibrosis-prone autoimmune diseases (*n* = 58), compared to the 165 healthy controls (FDR < 0.005) [16]. Interestingly, an overabundance of *Bifidobacterium* and *Lactobacillus*, which are known to be beneficial flora, was observed in patients with SSc [16,134]. Notably, patients with SSc showed a significant increase in Th17-inducing *Eggerthella lenta* strains and homocysteine-producing *C. bolteae* strains, indicating a potential microbiome-driven inflammation and vasculopathy in SSc [16].

#### 3.1.3. The GIT Manifestation-Related Gut Microbiome in SSc

Specific gut microbiota may be associated with the severity of GIT symptoms in patients with SSc, as evidenced by relatively high levels of *Lactobacillus*, *Blautia*, and *Coprococcus,* and relatively low levels of *Roseburia* and *Faecalibacterium* in patients with GIT symptoms compared to healthy controls [133]. At the species level, an increase in *Lactobacillus reuterii* and a decrease in the butyrate-producing species *Roseburia faecis* and *Faecalibacterium* (*F.*) *prausnitzii* were observed in patients with SSc with GIT symptoms [133]. In patients with SSc with low GIT symptom severity, *Clostridium*, *Blautia,* and *B. fragilis* were overabundant, whereas *Fusobacterium* was more abundant in patients with severe GIT symptoms [6,134] (Figure 3). *Lactobacillus* was high in patients with mild constipation, while *Parabacteroides* was higher in patients with severe constipation [6]. *Prevotella* was abundant in patients with SSc who had severe bloating/distension and diarrhea [6], while *Prevotella* (*P.*) *copri* was associated with worse GIT symptoms, such as fecal incontinence and malnutrition [135]. Notably, *Akkermansia* (*A.*) *muciniphila* was associated with severe diarrhea and fecal incontinence [135]. The fecal microbiome of patients with SSc with SIBO showed a relatively high proportion of *Bacteroides* spp. and *Uncl. Rickenellaceae* spp., and a relatively low proportion of *Uncl. Erysipelotrichacaea* spp. compared to healthy controls. However, no significant differences were reported between patients without SIBO and healthy controls [128]. Hence, to identify which microbial species represent the most effective targets to improve SSc pathology, comprehensive research is required to establish which species alleviate the highly heterogenous symptoms, including GI manifestation in SSc.

#### 3.1.4. Gut Microbial and Metabolic Interactions in SSc

The gut microbiota plays a role in regulating the host metabolic pathways through multidirectional chemical interactions between host signaling pathways and microbiota. Additionally, microbial metabolites, such as SCFAs and bile acids, influence the metabolic phenotype of the host and disease susceptibility [136]. Potential biological functions of metabolite-producing gut bacteria in the host have been demonstrated [137,138]. In patients with SSc, the gut microbiota showed increased pro-inflammatory noxious *Desulfovibrio*, which positively correlated with the level of alpha-N-phenylacetyl-l-glutamine and 2,4-dinitrobenzenesulfonic acid [5]. The pro-inflammatory microbial shift in the gut may be involved in the induction of intestinal damage and modulation of amino acid metabolism in the host [5]. A recent study reported an enhanced kynurenine pathway and a dysregulated urea cycle and lipid metabolisms, such as a down-regulation of tryptophan and up-regulation of kynurenine, dimethylarginine, and phenylacetylglutamine in plasma of patients with SSc [139]. These altered metabolic pathways in patients with SSc were associated with gut dysbiosis as well as inflammation, vasculopathy, and fibrosis [139]. However, the current data available regarding the microbial characteristics and metabolic profile of patients are lacking in the context of SSc. A better understanding of the microbial and metabolic networks in SSc will help in optimizing therapeutic strategies through targeted manipulation of gut microbiota.

### 3.2. Skin Microbiome in SSc

SSc characterized by skin thickening can be classified into limited cutaneous SSc (lcSSc) and diffuse cutaneous SSc (dcSSc) based on the extent of skin involvement [140]. In lcSSc, skin fibrosis is restricted to the hands, face, and distal extremities, whereas in dcSSc, fibrosis progresses more extensively, affecting the trunk and proximal extremities [2]. Although current research related to the skin microbiome in SSc is severely lacking, analysis of the microbiome associated with other inflammatory skin diseases, such as atopic dermatitis (AD), has provided valuable insights into the potential role of skin microbial communities in disease pathogenesis [141]. For example, skin *Staphylococcus* (*S.*) *aureus* was increased in patients with AD, and antimicrobial medication, such as bleach baths, improved symptoms and ameliorated the *S. aureus* burden [141].

Intact skin is crucial in maintaining tissue homeostasis and protecting tissues from infections, allergens, and environmental stimuli [142]. Commensal microorganisms on the skin’s surface produce biologically active metabolites that can kill pathogens. Moreover, commensals can stimulate keratinocytes and immune cells to protect our bodies from harmful and foreign substances. Notably, keratinocytes participate in skin homeostasis by recognizing microbes, especially PAMPs, through PRRs [143] and releasing immune mediators and antimicrobial peptides [144]. A defective skin epithelial barrier can lead to dysbiosis of skin microbiota, microbial translocation to the transepithelial area, colonization of opportunistic pathogens, immune cell activation, and tissue inflammation [142]. Indeed, the SSc epidermis exhibits increased antimicrobial peptide production, enhanced inflammatory reactions, and active TGF-β signaling in dermal fibroblasts [145].

A study related to the skin microbiome in SSc showed that the forearm skin of patients with early dcSSc exhibited higher *Rhodotorula glutinis* expression than that of healthy controls [146]. Moreover, the skin microbiome of patients with lcSSc or dcSSc contained a reduced abundance of lipophilic bacteria (*Propionibacterium*) and *Malassezia* fungus, as well as increased gram-negative bacteria (*Burkholderia*, *Citrobacter*, and *Vibrio*) [80]. Skin microbial dysbiosis is associated with a decreased lipid metabolism and an increased immune activation and TGF-β signaling. 

However, studies involving the characterization of the skin microbiome must be conducted with extreme caution, as the microbial community is markedly impacted by various factors including methods of specimen collection, biopsied skin areas, and sequencing methods [147]. Although routinely used dermal punch biopsy can easily obtain the epidermis and dermis, it is invasive. Moreover, different skin regions have their unique microbial community. Additionally, adopting strategies capable of differentiating between transient and resident bacteria is important in skin microbiome research [143]. Therefore, they should be included in the new technical analysis for studying the skin microbiome.

### 3.3. The Lung Microbiome in SSc

ILD and PH are the main causes of SSc-related mortality [4]. Although no studies have examined the role of lung microbes in SSc, the association of microbes in lung diseases, such as idiopathic pulmonary fibrosis (IPF), has been demonstrated. In the BAL of patients with IPF, an increased microbial burden and reduced microbial diversity were observed, which may be due to an increase in Firmicutes, *Streptococcus*, and *Veillonella* and a decrease in Proteobacteria [148,149]. Decreased microbial diversity was associated with declined lung function and high mortality [148,149]. Moreover, lung microbial dysbiosis was correlated with alveolar inflammation and disease progression in patients with IPF [150]. Previous reports support the hypothesis that the lung microbiome may be involved in disease onset, ILD severity, and pathological mechanisms, such as immune and fibroblast activation, in SSc [147]. 

The lungs were initially believed to be a sterile environment; however, the discovery of microbes in healthy lungs has garnered attention toward the lung microbiome [151]. The lungs are constantly exposed to diverse microorganisms via inhalation or micro-aspiration, which leads to communication with numerous microbes that are harbored in the mouth, oropharynx, and upper respiratory tract. In a study investigating microbial composition in the mouth, nose, lungs, and stomach of healthy subjects, the lungs were found to contain a unique microbiota, with only a portion of the species common between the lungs and mouth, suggesting that microbes that migrated from the mouth may be a source of the lung microbiome [152]. 

However, lung microbiome sequencing is technically challenging due to the relatively low microbial content in the lungs and contamination induced by the sampling method. The sampling of lower airways using bronchoscopy can cause pharyngeal contamination during the passage of the instrument through the nasal or oral cavity [151]. Therefore, a lung microbiome study requires advanced techniques to overcome the existing limitations.

## 4. Current and Future Therapeutic Strategies Targeting the Microbiome in SSc

Although medications for SSc are not yet established, clinical results support that the microbiome could be a possible therapeutic target. Probiotics and dietary modification may play a role in managing SSc-related GIT abnormalities. In this section, we review clinical trials targeting microbiomes in SSc to estimate the benefits and provide an optimal approach to microbiome challenges.

### 4.1. Probiotics and Fecal Microbiome Transplantation

Probiotics are live and non-pathogenic microbes that can provide health benefits [153]. Although the data on probiotic use in SSc is still lacking, some studies have reported the benefit of probiotics in alleviating SSc-associated GIT symptoms [154,155,156]. The use of probiotics Align (*Bifidobacterium* [*B.*] *longum sub. infantis*) and Culturelle (*Lactobacillus GG*) alleviated reflux and distention/bloating with a decreased total GIT symptom score in patients with SSc [154]. *Lactobacillus* (*L.*) *casei* improved reflux and abdominal distension [157], and probiotics complex (*L. paracasei*, *L. rhamnosus*, *L. acidophilus*, and *B. lactis*) reduced circulating Th17 cells in patients with SSc, suggesting immunomodulatory action of probiotics on SSc [155]. A four-month intervention with the multi-strain probiotic, Vivomixx (*Lactobacilli*, *Bifidobacteria*, and *Streptococcus*), demonstrated a good safety profile and significantly improved reflux symptoms in patients with SSc [158]. Furthermore, patients receiving fecal microbiome transplantation (FMT) using commercially available anaerobic cultivated human intestinal microbiota (ACHIM) showed improvements in bloating, diarrhea, and fecal incontinence [159,160]. A few clinical trials have been conducted and are currently underway to test the effect of the microbiota in SSc (Table 1).

*Lactobacillus* and *Bifidobacterium* are the most common genera for probiotics. The use of probiotics containing *Lactobacillus* and *Bifidobacterium* remains questionable, as patients with SSc demonstrate a unique gut microbial feature including an overabundance of *Lactobacillus* and *Bifidobacterium*. It is imperative to investigate whether an increase in *Lactobacillus* and *Bifidobacterium* is the cause or consequence of factors, such as medication (such as corticosteroids), diet, and supplements that can influence the GIT microbiome [161]. Despite concerns regarding the use of the two genera in SSc, probiotics containing them not only improved SSc-associated GIT manifestations but also were safe for patients. Additionally, the decrease in *Lactobacillus* in the oral cavity of patients with SSc has recently been identified, suggesting the protective role of *Lactobacillus* [162]. The evidence implies the potential of the two genera as therapeutic targets for the treatment of SSc. Further studies need to be performed to clarify the probiotic strains specific to treatment and their benefits and mechanisms on SSc.

However, some results have revealed potential risks of using probiotics, which may lead to side effects including systemic infection, excessive immune stimulation, and deleterious metabolic activity in susceptible individuals [163]. Particularly, probiotics may have negative effects on patients with immunocompromised conditions or serious illnesses. Therefore, SSc patients suffering from severe immunocompromised and digestive problems may need to be cautious about using probiotics. The safety and risks of probiotics should be thoroughly considered depending on the medical and disease status in SSc.

**Table 1:** Clinical trials to determine the efficacy of microbial therapeutics in SSc.

Interventions	Phase	Status	NCT Number	Outcome
ACHIM (10^9^ intestinal microbes/mL)	Phase 2	Recruiting	NCT04300426	Long-term safety and efficacy measures [160]
Vivomixx probiotics (*L. paracasei DSM 24733*, *L. plantarum DSM 24730*, *L. acidophilus DSM 24735*, *L. delbrueckii* subsp. *bulgaricus DSM 24734*, *B. longum DSM 24736*, *B. breve DSM 24732*, *B. infantis DSM 24737*, and *S. thermophilus DSM 24731*)	Phase 2	Unknown	NCT01804959	Improvement of GI reflux [158]
*L. paracasei*, *L. rhamnosus*, *L. acidophillus*, and *B. lactis*	Phase 3	Unknown	NCT02302352	No improvement of GI symptoms and a decrease in Th17 cells [155]
Culturelle *(Lactobacillus)*	Phase 4	Withdrawn	NCT01497743	–
ACHIM and cultivated medium	Phase 1 and 2	Completed	NCT03444220	Safety, decreased bloating, diarrhea, and fecal incontinence [159]
*Saccharomyces Boulardii* (SB) oral tablet and metronidazole (M)	Phase 4	Completed	NCT03692299	Decreased SIBO, diarrhea, abdominal pain, and bloating in the SB and M + SB groups [164]

### 4.2. Prebiotics and Dietary Modification

Prebiotics are defined as dietary fibers (starch, cellulose, inulin, and oligosaccharides such as galactooligosaccharides, fructooligosaccharides, and xylooligosaccharides) that are selectively utilized by host microbiota conferring health benefits [165,166]. Most prebiotics can promote the colonization of beneficial bacteria, including *Lactobacillus* and *Bifidobacterium* [165]. Several studies have shown that *Lactobacillus* and *Bifidobacterium* stimulated by prebiotics can inhibit the adhesion and growth of harmful bacteria by competing with pathogens for mucosal binding and exhibiting antagonistic effects through lactic acid or SCFAs produced by the two bacteria [167,168]. Dietary modification can also influence the GIT microbial communities and metabolite production [122]. A low-fat and high-fiber diet alleviated inflammation and intestinal dysbiosis in patients with ulcerative colitis [169]. In contrast, a high-fat diet reduced antimicrobial peptides and elevated the inflammatory cytokines, IFN-γ and TNF-α, in mice [170].

Although no studies have investigated the effect of dietary intervention on the SSc microbiome, few studies have demonstrated improvements in GIT symptoms by dietary interventions. A low-fermentable oligosaccharide, disaccharide, monosaccharide, and polyol (FODMAP) diet decreased diarrhea in patients with SSc presenting with fructose malabsorption [171] and improved GIT symptoms, as evaluated by a patient-reported outcome assessment [156]. Approximately 40% of patients with SSc suffer from fructose and lactose malabsorption [120], suggesting the importance of dietary modification in managing GIT symptoms. However, the data may not be sufficient to determine the role of dietary change in treating GIT symptoms due to a non-randomized controlled study. Further research requires long-term and randomized designs on dietary intervention.

The reduction in butyrate-producing bacteria is one of the characteristics of the SSc gut microbiome [16]. Although the benefits of diets promoting SCFA production in patients with SSc are unidentified, the trials may be putative therapeutic interventions [147].

### 4.3. Drug–Microbiota Combination Strategies for the Treatment of SSc

Despite some therapeutic advances, no specific therapies are available for SSc disease, and the optimal choice of therapeutics remains a controversial issue [140]. Medications contributing to symptomatic relief of SSc are sometimes associated with GIT dysfunction. Calcium channel blockers (CCB) and phosphodiesterase-5 (PDE-5) inhibitors are widely used to treat Raynaud’s phenomenon in patients with SSc. However, CCB (e.g., nifedipine and amlodipine) can worsen gastroesophageal reflux [172] and PDE-5 inhibitors (e.g., sildenafil) can inhibit antral and duodenal motor activity [173]. Recently, nintedanib, a tyrosine kinase inhibitor of PDGFR, FGFR, and VEGFR, is the first licensed drug to treat SSc-associated ILD [174]. Although nintedanib slows the decline in lung function, therapeutic approaches to reverse or stop disease progression are required. Furthermore, the most common adverse effect of nintedanib is GIT disorders, especially diarrhea and vomiting, which may lead to interruption or reduction in the effects of nintedanib [175]. Therefore, reducing GIT complications and recovering from a disease will be critical in SSc management. 

Recently, growing evidence suggests that gut microbiota can directly or indirectly participate in the treatment of diseases by modulating the bioavailability, efficacy, or toxicity of oral drugs [176]. Potential mechanisms by which gut microbiota can affect the bioavailability of oral drugs have been described as follows: (1) the alteration in GIT properties such as luminal pH, mucosal thickness, and transit time; (2) the influence on drug transport through the regulation of transporter genes by microbiota or microbial metabolites, or substrate competition between microbial components and drugs; and (3) the influence of microbial enzymes on drug metabolism [177]. For example, the probiotic *Escherichia coli* strain enhanced the absorption of the antiarrhythmic drug amiodarone, probably because it facilitated the ionization and mucosal diffusion of a weak base amiodarone by lowering intestinal pH [178]. Additionally, *Mycoplasma hyorhinis* expressed nucleoside analog-catabolizing enzymes that convert the chemotherapeutic drug gemcitabine into an inactive form, which could mediate the resistance of cancer cells to the drug [179].

Various studies have also reported the function of probiotic strains in alleviating drug-induced side effects [180]. Probiotics decrease chemo/immunotherapy-induced gastrointestinal toxicity such as colitis, mucositis, and diarrhea [181]. As a fundamental mechanism underlying the protective effects of probiotics on the gut, probiotics can enhance resistance to pathogens by competing with pathogenic bacteria for nutrients and inhibiting pathogenic bacterial adhesion to the gut epithelium [182] or by interfering with pathogen colonization through co-aggregation with pathogens [183]. Additionally, metabolites produced by probiotics (lactic acid, SCFAs, or bacteriocins) can suppress the growth of pathogens by lowering the pH of the lumen [184] and exhibit direct antibacterial activity [185], leading to microbial stabilization [180]. Representative probiotic strains, such as *Lactobacillus* and *Bifidobacterium,* present immunomodulatory properties and improve gut barrier function by suppressing pro-inflammatory cytokines, upregulating tight junction proteins (ZO-1, occludin, and claudin-1), and promoting mucin production [180,186]. Based on the beneficial functions of probiotics, the combination therapies of drugs and probiotics can be a promising therapeutic strategy in SSc by exerting potent anti-inflammatory and immunomodulatory effects, boosting drug activity, and reducing medication-induced adverse events. 

## 5. Experimental Mice Models as a Tool of the SSc Microbiome Study

A wide range of animal models has been developed to address the pathological mechanism of SSc [187]. However, microbiome study in SSc animal models is lacking and existing animal models cannot fully mimic the three main pathophysiological features of human SSc [188]. Microbiome-related animal studies are necessary to identify the potential role of the microbiome in the development of SSc and the possibility of applying microbiome-based therapy to SSc.

Although few studies have reported microbial dysbiosis in the SSc mouse model, one study uncovered the influence of gut microbial dysbiosis on the development of fibrosis in SSc-like mice induced by topoisomerase I peptide-loaded (TOPOIA) DC immunization [189]. The oral administration of streptomycin in early life induced gut microbial alterations, such as an increased Bacteroidetes/Firmicutes ratio and, consequently, aggravated skin and lung fibrosis in TOPOIA DC immunized mice. Recently, similar aberrations in the gut microbiota of patients with SSc and BLM-induced SSc mice were found [190]. The BLM-induced model is a commonly used inducible animal model which mimics systemic inflammation and autoimmunity in the early stages of SSc [191]. Gut microbiota in patients with SSc and BLM mice are characterized by decreased Bacteroidetes and increased Firmicutes at the phylum level, as well as elevated *Lactobacillus* at the genus level, suggesting that the BLM model can likely represent pathological gut dysbiosis of patients with SSc. Additionally, oral administration of the bacterial metabolite butyrate alleviated skin and lung fibrosis in BLM mice, presenting the possibility of microbiome targeting for SSc treatment [192].

Chronic sclerodermatous graft-versus-host disease (Scl-GVHD) mice resemble human SSc, as the clinical forms of GVHD mice include inflammation and fibrosis of the liver, kidney, and GIT, as well as the skin and lungs [193]. Thus, GVHD mice may be a potential animal model for studying and targeting gut microbiome and GIT symptoms. Characterizing the microbiome in various SSc mouse models exhibiting inflammatory, vascular, and fibrotic phenotypes will be a powerful tool to understand the intricate association between aberration in gut microbiota and pathological features of SSc to devise disease-modifying agents.

Importantly, gut microbiota variability in mouse models leads to poor reproducibility of data. Multiple factors such as source, facility, diet, husbandry, and delivery can modulate the gut microbiota of experimental animals [194]. Therefore, in studies on the gut microbiome of disease mouse models, including SSc, strict steps must be taken to control and reduce these variables in experimental animals.

## 6. Future Prospects for SSc Microbiome Research

Although studies to establish the microbiome profiles of heathy subjects are currently undergoing, numerous microorganisms and their function remain unknown in human health. Additionally, the high inter-individual diversity of the gut microbiome is a major barrier to developing microbiome-modifying applications. Expecting a common mechanistic pathway and predictable effect of the microbiome-based application in SSc with different and multiple disease phenotypes may be difficult. Therefore, for the application of SSc microbiome-based therapy, research on the microbiome as personalized medicine should ultimately be conducted.

Currently, microbiome research is moving toward collectively identifying bacterial taxa in interacting organs, including blood [195]. A comprehensive understanding of microbial changes is of great importance in SSc, which presents pathological manifestations in various organs such as the skin, lungs, and intestines. Thus, multi-omics technologies combined with bioinformatics are essential for defining disease-specific microbiome profiles, as well as multidirectional interactions between the microbiome and the host in SSc. Advances and utilization of these technologies will enable the determination of proper microbial targets and improve microbiome research in SSc, leading to the feasibility of personalized medicine.

## 7. Conclusions

SSc is a heterogenous disease with diverse cellular and molecular abnormalities, which presents challenges in understanding its pathophysiology and development of potential therapeutics. Although a combination of genetic and environmental factors is associated with the etiology of SSc, the pathogenesis of SSc is not completely known. However, increasing evidence indicates that SSc is associated with microbial dysbiosis with unique features and GIT is affected in most of the patients with SSc. The GIT microbiome of patients with SSc is characterized by a reduction in commensals, including butyrate-producing bacteria, and an increase in pathobionts. The microbiome is probably one of the key factors in SSc pathogenesis. Currently, microbiome-based applications in patients, as well as specific therapies for SSc, are lacking. However, continued progress in characterizing the microbiome will provide valuable insights into the molecular pathways underlying SSc pathogenesis that will inform the development of effective therapeutic strategies. As a future advanced medication, drug–microbiota combination therapy has the potential to contribute to the treatment and management of SSc in terms of reducing side effects and enhancing drug efficacy. Moreover, well-defined animal models and microbiome studies coupled with multi-omics analyses are required to discover microbial targets and mechanisms for future SSc therapy.

## Data Availability

The data presented in this study are available in the article.
